# Angiotensin-Converting Enzyme Inhibitory Activity of Selected Phenolic Acids, Flavonoids, Their *O*-Glucosides, and Low-Molecular-Weight Phenolic Metabolites in Relation to Their Oxidation Potentials

**DOI:** 10.3390/metabo15070443

**Published:** 2025-07-01

**Authors:** Danuta Zielińska, Małgorzata Starowicz, Małgorzata Wronkowska, Henryk Zieliński

**Affiliations:** 1 Department of Chemistry, University of Warmia and Mazury in Olsztyn, Plac Łódzki 4, 10-721 Olsztyn, Poland; danuta.zielinska@uwm.edu.pl; 2 Team of Chemistry and Biodynamics of Food, Institute of Animal Reproduction and Food Research, Polish Academy of Sciences, Trylińskiego 18, 10-683 Olsztyn, Poland; m.starowicz@pan.olsztyn.pl (M.S.); m.wronkowska@pan.olsztyn.pl (M.W.)

**Keywords:** phenolic acids, flavonoids, glucosides, phenolic metabolites, ACE inhibitory activity, differential pulse voltammetry, antioxidant power

## Abstract

**Background/Objectives**: In this study, the angiotensin-converting enzyme (ACE) inhibitory activity of selected phenolic acids, flavonoids, their *O*-glucosides, and low-molecular-weight phenolic metabolites was addressed to show their importance against blood hypertension. **Methods**: A fluorescence assay was used for the determination of the ACE inhibitory activity, whereas the first anodic peak oxidation potential ($E_{pa}$) was provided by the differential pulse voltammetry (DPV) method. The relationship between the ACE inhibitory activity and $E_{pa}$ was evaluated. **Results**: Phenolic acids showed a very low ACE inhibitory activity, and their rank was chlorogenic acid > p-coumaric acid > sinapic acid > gentisic acid > ferulic acid > syringic acid > vanillic acid > protocatechuic acid > caffeic acid. The low-molecular-weight phenolic metabolites of flavonoids showed a moderate ACE inhibitory activity. In contrast, flavonoid aglicones had the highest ACE inhibitory activity, and the order was luteolin > quercetin > kaempferol > cyanidin > delphinidin > pelargonin > naringenin. A lower inhibition activity was noted for quercetin-3-*O*-glucoside, luteolin-4′-*O*-glucosides, cyanidin-3-*O*-glucoside, and pelargonidin-3-*O*-glucosides, whereas a higher ACE inhibition activity was observed for 7-*O*-glucosides of luteolin, apigenin, and kaempferol. A lack of correlation was found between the IC_50_ of phenolic acids, low-molecular-weight phenolic metabolites, and their $E_{pa}$ values. In contrast, weak positive correlations were found between the IC_50_ of aglicons, 3-*O*-glucosides, 7-*O*-glucosides, and their $E_{pa}$ values provided by the DPV (r = 0.61, r = 0.66 and r = 0.88, respectively). **Conclusions**: This study expands our knowledge of the ACE inhibitory activity of phenolic compounds.

## 1. Introduction

Cardiovascular disease (CVD) is closely related to high blood pressure [1]. The regulation of blood pressure involves pharmacological therapies and non-pharmacological management or lifestyle changes following a healthy diet [2]. Angiotensin-I converting enzyme (ACE) plays a key physiological role in the control of blood pressure in the rennin–angiotensin system [3]. ACE converts the inactive decapeptide, angiotensin I, to the potent vasopressor octapeptide, angiotensin II, and inactivates bradykinin [4]. Elevated blood pressure is often treated with synthetic drugs that inhibit ACE [5], but synthetic ACE inhibitors might be accompanied with unwanted side effects [6,7]. Alternative inhibitors from natural sources are still being sought, because they may have a better profile as drugs and cause fewer side effects [8]. Recently, a lot of bioactive compounds with potent ACE inhibitory activity have been characterized from enzymatic hydrolysates of casein [9], tuna bone protein [10], soybean [11], wheat germ [12], lentil [13], apricot almond meal hydrolysate [14], and many other foods [15,16].

The ACE inhibitory activity in foods of plant origin could be caused by a synergistic effect of flavonoids or from other unidentified components in the extracts, since the ACE inhibitory activity of flavonoids isolated from other plants has been reported previously [17,18]. Both phenolic acids and flavonoids have been reported as dietary modulators of cardiovascular function by the regulation of blood pressure [19], oxidative stress [20], and inflammation [21] in the cardiovascular system; however, their antihypertension activity has been scarcely investigated in relation to their antioxidant potential. Although it is known that the intestinal microflora participates in the metabolism of flavonoids [22,23,24,25], there is a lack of information about the specific properties of these metabolites. Several studies have shown that the metabolic activity of the gut microflora transforms polyphenols into phenolic acid metabolites such as phenylvaleric, phenylpropionic, phenylacetic, benzoic, and hippuric acids [24,26], which is related to the phenolic compound being metabolized [27,28]. Some of these compounds might be biologically active, be absorbed, and contribute to the biological activity of dietary flavonoids. Quercetin, as well as rutin (quercetin rutinoside), could be metabolized to 3-(3,4-dihydroxyphenyl)propionic acid via ring fission of the C ring in various ways, and this could be subsequently degraded into 3,4-dihydroxyphenylacetic acid [29,30]. The latest one may also be dehydroxylated to m- or p-hydroxyphenylacetic acid, phenylacetic acid, and 3-methoxy-4-hydroxyphenylacetic acid (homovanillic acid), which has been previously reported in the literature as a biomarker for microbial and/or hepatic metabolism of quercetin [31,32].

The chemical structure and the number and position of hydroxyl groups determine the antioxidant potential, which may be the reason for the observed inhibitory effect on ACE activity. This relationship has been previously described for the major green and black tea catechins [33,34] and hydroxybenzoic acids, differing only in the number of hydroxyl groups on the benzene ring. Studies have shown that phenolic compounds inhibit ACE via interaction with the zinc ion, which is stabilized by other interactions with amino acids in the active site [35]. The antioxidant properties of phenolic acids, flavonoids, their *O*-glucosides, and low-molecular-weight phenolic metabolites may be evaluated by electrochemical methods such as cyclic voltammetry (CV), differential pulse voltammetry (DPV), and square wave voltammetry (SWV), which are based on electron transfer [36,37]. CV is a versatile electrochemical technique in which the potential of a working electrode is cyclically swept between defined limits while recording the resulting current [38,39,40]. Usually, voltametric data provide qualitative information (peak of anodic oxidation potential (E_pa_) indicating antioxidant strength) and quantitative information (peak currents or charges proportional to antioxidant concentration). A low value of E_pa_ corresponds to high antioxidant power [41]. The DPV is an excellent technique to determine the E_pa_, as a scan takes seconds and can provide valuable information on the electroactivity of sample or compounds in one run if the peaks are distinguishable [42,43]. DPV requires minimal sample preparation—an aqueous or hydroalcoholic extract/standard solutions mixed with a supporting electrolyte.

The aim of this study was (1) to investigate the ACE inhibitory activity of phenolic acids, flavonoids, their main glucosides, and the low-molecular-weight phenolic metabolites of quercetin and rutin; and (2) to determine the relationship to their antioxidant power characterized by the anodic peak potentials (E_pa_) provided by the DPV technique.

## 2. Materials and Methods

### 2.1. Chemicals

Captopril was obtained from Sigma-Aldrich (No C4042, St. Louis, MO, USA). Angiotensin-converting enzymes (ACE) from porcine kidneys (EC 3.4.15.1) were purchased from Sigma-Aldrich (St. Louis, MO, USA). A three-tris-base buffer was as follows: buffer A: 300 mM Tris-base buffer (pH 8.3) with 2 mM ZnCl_2_; buffer B: 150 mM Tris-base buffer (pH 8,3); and buffer C: 150 mM Tris-base buffer (pH 8.3) with 1125 M NaCl. The substrate *o*-aminobenzoylglycyl-*p*-nitorphenylalanylproline (Abz-Gly-Phe(NO_2_)-Pro) was obtained from BACHEM (Bubendorf, Switzerland). Standards of phenolic acids, flavonoids, glucosides, and low-molecular-weight phenolic metabolites were obtained from Sigma-Aldrich (St. Louis, MO, USA) and Extrasynthese (Genay, France). Water was purified using the Mili-Q system (Milipore, Bedford, MA, USA).

### 2.2. Stock Solutions of Standards of Phenolic Acids, Flavonoids, Their O-Glucosides, and Low-Molecular-Weight Phenolic Metabolites for ACE Inhibitory Activity Determination

Stock solutions at 1 mM of selected phenolic acids, flavonoids, their O-glucosides, and low-molecular-weight phenolic metabolites were prepared in 80% methanol. The concentration was confirmed by a UV measurement according to Franke et al. [44]. A wide range of concentrations of each compound was prepared by serial dilutions in deionized water to determine the IC_50_ value. The control compounds used were captopril solution (0.1 μM solution) and reduced glutathione (GSH; 1 mM solution), prepared in deionized water. The ACE inhibitory activity was obtained from three repetitions (*n* = 3) for each concentration.

### 2.3. Angiotensin-I-Converting Enzyme Inhibitory Assay

Angiotensin-converting enzyme (ACE) activity inhibitory assay was performed according to the method of Sentandreu and Toldra [45]. The procedure was based on the hydrolysis of the internally quenched substrate *o*-aminobenzoylglycyl-*p*-nitorphenylalanylproline (Abz-Gly-Phe(NO_2_)-Pro) by the reaction of ACE. The fluorescence generated by the liberation of the product (the *o*-aminobenzoylglycine group) was read in a microplate multiscan fluorometer. The production of fluorescence was inhibited by the presence of functional compounds with an ACE inhibitory effect. The fluorescence was measured immediately after mixing (0 min) and then after 30 min of reaction at an excitation of 365 nm and an emission of 405 nm. To calculate the % of inhibition of ACE, the following equation was used:

Relative ACE activity% = 100 − (ΔRFU_sample_ × 100/ΔRFU_negative control_),

where ΔRFU = RFU at time 30 – RFU at time 0.

The wide range of concentrations of each compound prepared by serial dilutions in deionized water was used for the determination of the IC_50_ value, indicating the sample concentration at 50% inhibition of ACE activity calculated by the linear regression analysis of logarithmic plots.

### 2.4. Measurement of the Anodic Oxidation Potentials of Phenolic Acids, Flavonoids, Their O-Glucosides, and Low-Molecular-Weight Phenolic Metabolites with Differential Pulse Voltammetry

A potentiostat/galvanostat G 750 (Gamry Instruments, Warminster, USA) was used for voltametric experiments. A conventional three-electrode system: a 3 mm diameter glassy carbon working electrode (BAS MF-2012), an Ag/AgCl electrode as a reference electrode, and a platinum as counter electrode, was used in the study. Differential pulse voltametric experiments were performed with phenolic acids and flavonoids prepared in a 1:1 (*v*/*v*) mixture of 500 µM standard solution and 0.2 M Britton-Robinson (B-R) buffer (pH 6.0 in 80% methanol), which also served as a supporting electrolyte [46]. The voltametric experiments were performed at room temperature using an electrochemical cell (volume 200 µL), to which the analyzed standard solution, mixed previously with the buffer solution, was introduced. The differential pulse voltammograms were acquired in the range of −100 to 1300 mV at a scanning rate of 100 mV/s. Before use, the surface of the glassy carbon electrode was carefully polished with 0.05 µm alumina paste, ultrasonically rinsed in deionized water, and then washed with methanol. This procedure was repeated after each cycle. For test purposes, the first anodic oxidation potential was recorded (mV).

### 2.5. Statistical Analysis

Results are given as the average ± standard deviation (SD). One-way analysis of variance (ANOVA) was used for the analysis of significant differences in the multifaceted biological activities of quercetin and its glucosides (*p* < 0.05) (GraphPad Prism version 9 for Windows, GraphPad Software, San Diego, CA, USA). The correlation analysis was performed, and the Pearson correlation coefficient was calculated.

## 3. Results

### 3.1. ACE Inhibitory Activity and the First Oxidation Potential of Phenolic Acids

**Figure 1:**
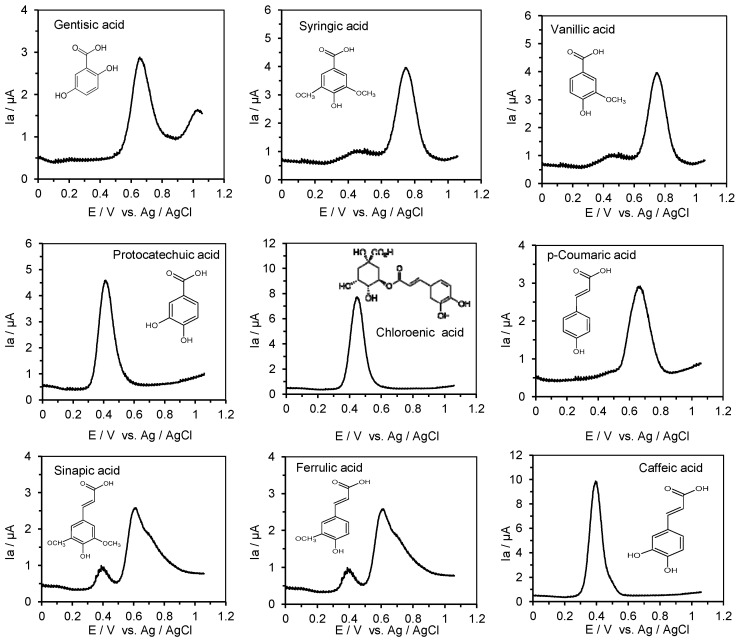
Differential pulse voltammograms of 0.25 mM of standard solutions of benzoic acid derivatives (gentisic, syringic, vanillic, homovanillic, and protocatechuic acid) and cinnamic acid derivatives (chlorogenic, p-coumaric, sinapic, ferulic, and caffeic acid) in 0.1 M Britton-Robinson (B-R) buffer, pH 6.0 in 80% methanol, recorded from −0.1 to +1.2 V; scan rate 100 mV s^−1^.

The results obtained for the ACE inhibitory activity of selected phenolic acids are shown in Table 1, whereas the differential pulse voltammograms are provided in Figure 1.

Phenolic acids showed a very low ACE inhibitory activity. The IC_50_ values obtained for phenolic acids ranged between 134 (chlorogenic acid) and 9105 µM (caffeic acid). The IC_50_ value represents the concentration of each compound that inhibits ACE activity by 50%. A lower IC_50_ value indicates higher ACE inhibitory activity. The IC_50_ values of phenolic acids were significantly different (*p* < 0.05 (Table 1). The first oxidation peak potentials of the benzoic acid derivatives ranged from 417 mV (protocatechuic acid) to 748 mV (vanillic acid), whereas cinnamic acid derivatives showed values from 397 mV (caffeic acid) to 672 mV (p-coumaric acid) (Table 1). A lower value of oxidation potential indicates higher antioxidant activity of the compound.

### 3.2. ACE Inhibitory Activity and the First Oxidation Potential of Flavonoids and Their O-Glucosides

**Figure 2:**
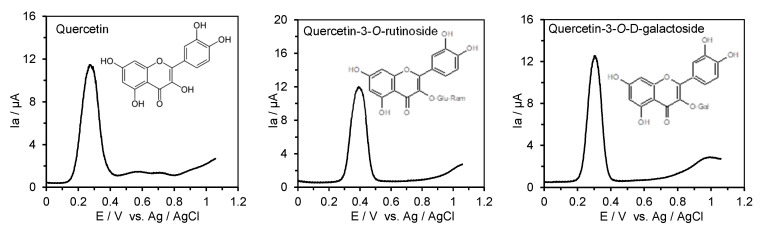

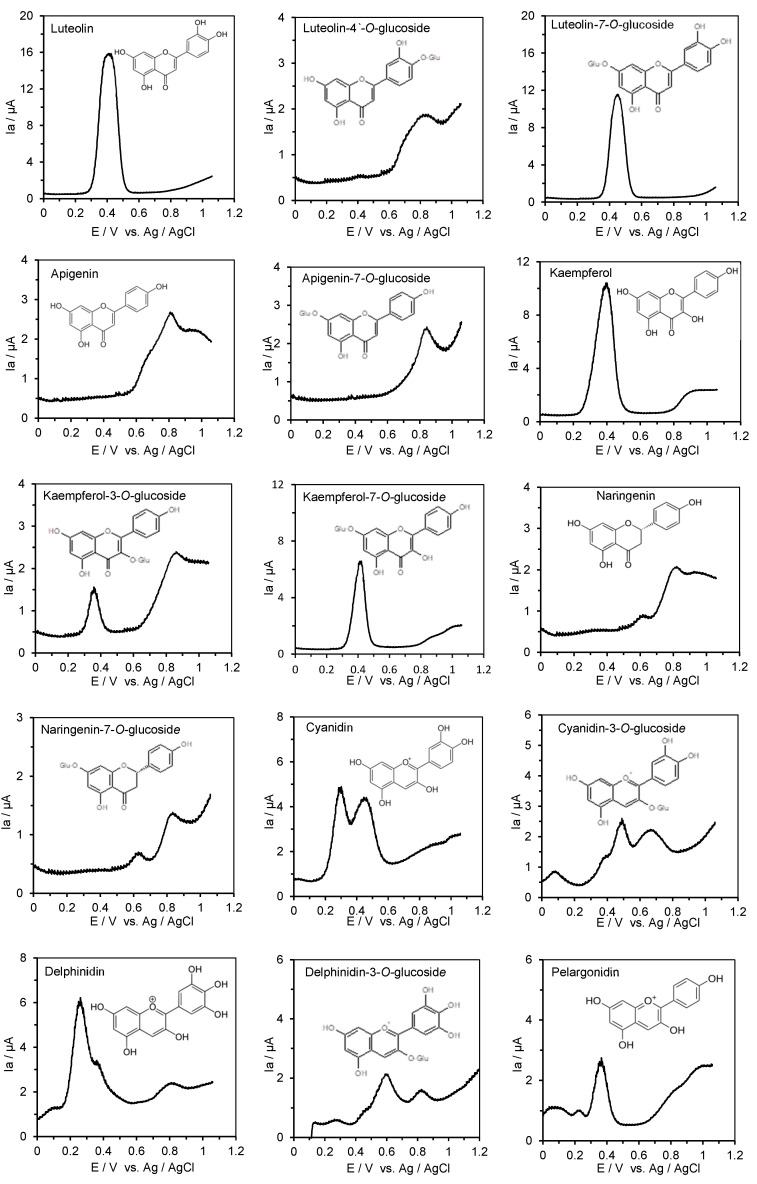

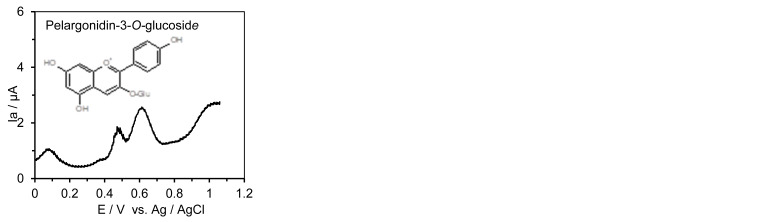
Differential pulse voltammograms of 0.25 mM of standards solution of rutin, quercetin, luteolin, apigenin, kaempferol, naringenin, cyanidin, delphinidin, and pelargonin and their main *O*-glucosides in 0.1 M Britton-Robinson (B-R) buffer, pH 6.0 in 80% methanol, recorded from −0.1 to +1.2 V; scan rate 100 mV s^−1^.

The ACE inhibitory activity of selected flavonoids and their *O*-glucosides, as well as the antioxidant potential of these compounds described by the value of the first oxidation potential provided by the DPV technique, are shown in Table 2, whereas the recorded differential pulse voltammograms of standards solution of rutin, quercetin, luteolin, apigenin, kaempferol, naringenin, cyanidin, delphinidin, and pelargonin and their the main *O*-glucosides in 0.1 M Britton-Robinson (B-R) buffer, pH 6.0 in 80% methanol, are presented on Figure 2.

In this study, the IC_50_ values for the angiotensin-I-converting enzyme inhibition of selected flavonoids and their glucosides ranged from 59.3 µM for quercetin-3-*O*-rutinoside to 390.8 µM for naringenin in comparison to glutathione (IC_50_ = 41.7 µM) and captopril (IC_50_ = 0.0059 µM). The IC_50_ values of selected flavonoids and their glucosides were significantly different (*p* < 0.05) based on the one-way analysis of variance (ANOVA) (Table 2). The order of the ACE enzyme inhibitory activity of non-glucosides selected flavonoids was luteolin > quercetin > kaempferol > cyanidin > delphinidin > pelargonin > naringenin, thus indicating the importance structure–activity relationship. The IC_50_ of luteolin was fivefold lower than that value for naringenin, thus simply indicating that the ACE enzyme inhibitory activity of luteolin was five times higher. In this study, higher values of the IC_50_ of hyperoside (quercetin-3-*O*-glucoside), luteolin-4′-*O*-glucosides, cyanidin-3-*O*-glucoside, and pelargonidin-3-*O*-glucoside by 30, 15, 5, and 29% were noted as compared to the IC_50_ of the respective aglycon. In this study, the ACE inhibitory activity of selected flavonoids and their *O*-glucosides was between two and tenfold lower than that determined for small tripeptide reduced glutathione (GSH). Moreover, the range of ACE inhibitory activity of phenolic acids (134–9105 µM) and flavonoids and their glucosides (59.3–390.8 µM) indicates that flavonoids and their glucosides exhibit higher ACE inhibitory activity from 2 to 23 times, respectively. It should be pointed out that the ACE inhibitory activity of chlorogenic, p-coumaric, and sinapic acid was within the range shown for flavonoids and their glucosides.

The order of the antioxidant potential of flavonoids and their glucoside based on the value of the first oxidation potential indicates that pelargonin, cyanidin, and pelargonidin can be considered as compounds withhigh antioxidant power (E_p_ < 0.3 V), whereas the remaining compounds can be classified as moderate antioxidants (0.3 V < E_p_ < 0.8 V). This order was drawn according to the work by Blasco et al. [47], in which differentiation of the antioxidant power of phenolic compounds was based on values of their oxidation potentials, thus supporting current knowledge on the structural features and the nature of substitutions on rings B and C determining the antioxidant activity of flavonoids [48].

### 3.3. ACE Inhibitory Activity and the First Oxidation Potential of Low-Molecular-Weight Phenolic Metabolites of Quercetin and Rutin

**Figure 3:**
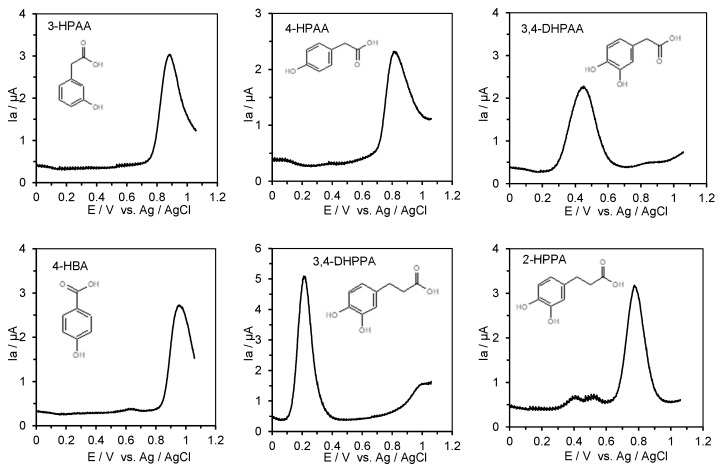

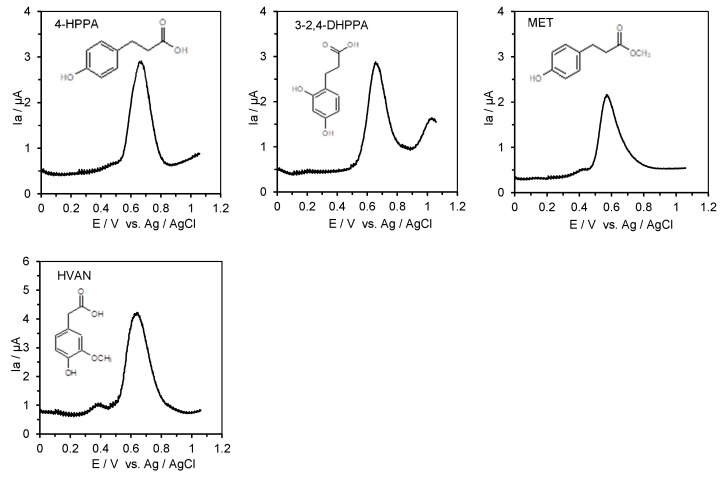
Differential pulse voltammograms of 0.25 mM of standard solution (final concentration) of the low-molecular-weight phenolic metabolites in Britton-Robinson (B-R) buffer; pH 6.0 in 80% methanol recorded from −0.1 to +1.2 V; scan rate 100 mV s^−1^.

The ACE inhibitory activity of the low-molecular-weight phenolic metabolites of quercetin and rutin, together with the antioxidant potential of these compounds, described by the value of the first oxidation potential provided by the DPV technique, are shown in Table 3, whereas the recorded differential pulse voltammograms of the standard solutions of these compounds in 0.1 M Britton-Robinson (B-R) buffer, pH 6.0 in 80% methanol, are provided on Figure 3.

The low-molecular-weight phenolic metabolites of flavonoids showed moderate ACE inhibitory activity. The obtained IC_50_ values ranged between 490 (µM) (4-HPPA) and 1461 (µM) (HVA). The IC_50_ values of phenolic metabolites were significantly different (*p* < 0.05) (Table 3). A lower IC_50_ value indicates higher ACE inhibitory activity. In our study, the ranking of ACE inhibitory activity found was as follows: captopril >>> GSH > 4-HPPA > 3,4-DHPPA > 3,4-DHPAA > 3-HPAA > MET > 3-2,4-DHPPA > 4-HPAA > HVA. The ACE inhibitory activity of 4-HPPA was almost 12-fold lower than the IC_50_ of small tripeptide reduced glutathione (GSH), whereas the ACE inhibitory activity of HVA was about 35-fold lower in relation to GSH. The first oxidation peak potentials of the low-molecular-weight phenolic metabolites ranged from 218 mV (3,4-DHPPA) to 883 mV (3-HPAA). A lower value of oxidation potential indicates higher antioxidant activity of the phenolic metabolite. Therefore, taking into account the values of the first oxidation potential of the studied metabolites, only the 3-(3,4-dihydroxyphenyl)propionic acid (3,4-DHPPA) could be considered as compounds with high antioxidant power (0.3 V < Epa), whereas MET, HVA, 3-2,4-DHPPA, 4-HPPA, and 2-HPPA had moderate antioxidant power (0.3 V < Epa < 0.8 V). In contrast, 3-hydroxyphenylacetic acid (3-HPAA) and 4-hydroxyphenylacetic acid (4-HPAA) showed weak antioxidant power (Epa ˃ 0.8 V). This conclusion was drawn according to the work by Blasco et al. [47], in which differentiation of the antioxidant power of phenolic compounds was based on the values of their oxidation potentials.

## 4. Discussion

The results provided in this study of the ACE inhibitory activity of phenolic acids followed the data provided by Shukor et al. [35], who showed ACE inhibition with IC_50_ values ranging from 2 to 9.3 mM. In contrast, captopril, which is a synthetic drug, showed extremely high activity. Benzoic acid derivatives’ inhibition with IC_50_ values ranged from 0.834 (gentisic acid) to 6.235 mM (protocatechuic acid), whereas higher ACE inhibitory activity was found for cinnamic acid derivatives with IC_50_ values ranging from 0.134 (chlorogenic acid) to 0.980 mM (ferulic acid) with exception of the very low ACE inhibitory activity of caffeic acid. Previously, Zielinska et al. [49] showed a low ACE inhibitory activity of caffeic acid (IC_50_ = 8.97 ± 0.08 mM), and this value corresponded to that provided in this study (IC_50_ = 9.11 ± 0.06 mM). The ACE inhibitory activity of caffeic acid was about 120-fold lower than that determined for the small tripeptide reduced glutathione (GSH). The ranking of ACE inhibitory activity found in this study was as follows: captopril > GSH > chlorogenic acid > p-coumaric acid > sinapic acid > gentisic acid > ferulic acid > syringic acid > vanillic acid > protocatechuic acid > caffeic acid. The ACE inhibitory activity of chlorogenic acid was threefold lower than the IC_50_ of small tripeptide reduced glutathione (GSH), whereas the ACE inhibitory activity of caffeic acid was about 120-fold lower in relation to GSH. These results indicate that the food source of cinnamic acid derivatives may play some role in ACE inhibition as compared to the benzoic acid derivatives. Taking into account the values of the first oxidation potential of the studied compounds, both groups of phenolic acids can be ranked as compounds with moderate antioxidant strength (0.3 V < Epa < 0.8 V). This conclusion was drawn according to the work by Blasco et al. [48], in which differentiation of the antioxidant power of phenolic compounds was based on values of their oxidation potentials. Having this finding, the question regarding the impact of the antioxidant potential of phenolic acids on their ACE inhibitory activity was addressed. In this study, a lack of correlation was found between the IC_50_ of phenolic acids and their anodic oxidation potentials provided by differential pulse voltammetry. The correlation coefficient had a value r = −0.47, thus clearly indicating that factors other than antioxidant activity may affect the ACE inhibitory activity of the phenolic acids. Docking studies indicated that phenolic acids inhibit ACE via interaction with the zinc ion, and this interaction is stabilized by other interactions with amino acids in the active site [35]. For the hydroxybenzoic acids and hydroxycinnamic acids, with a different number of hydroxyl groups on the benzene ring, an increase in ACE inhibitory activity with increasing numbers of hydroxyl groups was observed. The ACE-inhibiting activity of phenolic acids is also correlated with another functional group—the methoxy group. In this study, it was noted that the IC_50_ of ferulic acid was 10 times lower than the IC_50_ of caffeic acid.

The order of the ACE enzyme inhibitory activity of selected flavonoids (luteolin > quercetin > kaempferol > cyanidin > delphinidin > pelargonin > naringenin) indicates the importance of the structure–activity relationship. It was found that the substitution of glucose at position −3 at the ring C or 4′ at the ring B slightly decreased the ACE inhibitory activity of these compounds. Zielińska et al. [50] determined the ACE inhibitory activity of the marker polyphenols in apples, e.g., phloretin and phloridzin (phloretin 2′-*O*-glucose), belonging to the chemical class of dihydrochalcones. Phloridzin consists of a C_6_–C_3_–C_6_ skeleton structure (two aromatic rings connected by a C3 chain) with a β-D-glucopyranose moiety attached at position 2′. The phloretin showed IC_50_ = 286.83 ± 2.54 µM, being 25% higher than the IC_50_ of phloretin 2′-*O*-glucose [45]. This finding suggests that the β-D-glucopyranose moiety attached at position 2′ may also decrease the ACE inhibitory activity. However, in this study, exceptions were found in relation to rutin (quercetin-3-*O* rutinoside), kaempferol-3-*O*-glucoside, and delphinidin-3-*O* glucoside, where lower IC_50_ by 50, 16, and 16% were noted in relation to aglicones. Moreover, IC_50_ values of 7-*O*-glucosides of luteolin, apigenin, kaempferol, and naringein were also decreased by 20, 35, 26, and 9% in comparison to respective aglycons, thus clearly indicating that the substitution of glucose at position −7 at ring C, as well as those shown as exceptions, enhance the ACE inhibitory activity of these compounds. Moreover, structure–activity relationship studies have shown that the combination of sub-structures on the flavonoid skeleton that increase ACE inhibitory activity is made up of the following elements: (a) the catechol group in the B-ring, (b) the double bond between C2 and C3 at the C-ring, and (c) the ketone group in C4 at the C-ring [35]. These findings follow our observation that the substitution position in the flavonoid basic structure may affect the ACE inhibitory activity, which, in some cases, may decrease or increase, as we showed for 7-*O*-glucosides, likely reported for the first time in this study. Guerrero et al. [51] evaluated the in vitro ability of 17 flavonoids belonging to five structural subtypes to inhibit ACE in order to establish the structural basis of their bioactivity. The high ACE inhibitory activity provided by the same fluorometric method as in our study showed luteolin, quercetin, rutin, kaempferol, rhoifolin, and apigenin with IC_50_ values of 23, 43, 64, 178, 183, and 196 µM, respectively. These IC_50_ values are comparable with those provided in this study. Using data on oxidation potentials, the question regarding the impact of the antioxidant activity of flavonoids and their glucosides on their ACE inhibitory activity was addressed. In this study, weak positive correlations were found between the IC_50_ of aglicons, 3-*O*-glucosides, 7-*O*-glucosides, and their anodic oxidation potentials provided by differential pulse voltammetry. The correlation coefficients had r = 0.61, r = 0.66, and r = 0.88, respectively, thus indicating that the antioxidant activity related to the position of glucose substitution on rings B and C may affect the ACE inhibitory activity of flavonoid glucosides. This finding is in contrast to the relationship between the ACE inhibitory activity of the phenolic acids and the values of their oxidation potentials.

The ACE inhibitory activity of low-molecular-weight phenolic metabolites of flavonoids is presented for the first time. Previously, Giménez-Bastida [52] showed strong inhibitory activity of 3,4-DHPAA against the formation of advanced glycation end products, thus suggesting the multifaceted activity of low-molecular-weight phenolic metabolites. The following various phenolic acids have been reported as degradation products of quercetin by colonic microbiota: 2-(3,4-dihydroxyphenyl) acetic acid, 2-(3-hydroxyphenyl)acetic acid, and 3,4-dihydroxybenzoic acid (protocatechuic acid), together with 2-(3-methoxy-4-hydroxyphenyl)acetic acid (homovanillic acid), 3-methoxy-4-hydroxybenzoic acid (vanillic acid), 3-(3,4-dihydroxyphenyl)propionic acid, and 3-(3-hydroxyphenyl) propionic acid [48,53,54]. The same or similar metabolites have also been reported for other flavonoids [53,54]. Recent studies have suggested the potential role of some phenolic antioxidants in inhibiting ACE activity [51,55,56]; however, no information exists on the ACE inhibitory activity of low-molecular-weight phenolic metabolites. The range of ACE inhibitory activity of low-molecular-weight phenolic metabolites (490–1461 µM) and flavonoids and their glucosides (59.3–390.8 µM) indicates that flavonoids and their glucosides exhibit higher ACE inhibitory activity by eight and four times, respectively. However, when a comparison was done for the range of ACE inhibitory activity of phenolic acids (134–9105 µM), the ACE inhibitory activity of phenolic metabolites was about four times higher in relation to the benzoic acid derivatives and on a comparable level in relation to the cinnamic acid derivatives. These results indicate that low-molecular-weight phenolic metabolites of flavonoids may have some beneficial effects in ACE inhibition. However, the question regarding the impact of the antioxidant potential of phenolic metabolites on their ACE inhibitory activity was addressed. A lack of correlation was found between the IC_50_ of phenolic metabolites and their anodic oxidation potentials provided by the differential pulse voltammetry. The correlation coefficient had a value r = 0.28, thus clearly indicating that factors other than the antioxidant power of the phenolic metabolites may affect the ACE inhibitory activity of the phenolic acids. These findings are in agreement with the same conclusion drawn for phenolic acids.

The graphical presentation of the IC_50_ of ACE inhibitory activity of phenolic compounds is provided in Figure S1 (please see Supplementary Materials). In summary, quercetin, quercetin rutinoside, luteolin and its glucosides, kaempferol and its glucosides, followed by low-molecular-weight phenolic metabolites showed the highest ACE inhibitory activity. This study expands our knowledge of the ACE inhibitory activity of phenolic compounds and their metabolites.

## 5. Conclusions

Phenolic acids showed very low ACE inhibitory activity, and their rank was chlorogenic acid > p-coumaric acid > sinapic acid > gentisic acid > ferulic acid > > syringic acid > vanillic acid > protocatechuic acid > caffeic acid. The low-molecular-weight phenolic metabolites of flavonoids showed moderate ACE inhibitory activity. In contrast, higher ACE inhibitory activity was noted for flavonoids and their glucosides. The order of the ACE enzyme inhibitory activity of flavonoid aglicones was luteolin > quercetin > kaempferol > cyanidin > delphinidin > pelargonin > naringenin, whereas lower inhibition activity was noted for quercetin-3-*O*-glucoside, luteolin-4′-*O*-glucosides, cyanidin-3-*O*-glucoside, and pelargonidin-3-*O*-glucosides. In contrast, higher ACE inhibition activity was observed for 7-*O*-glucosides of luteolin, apigenin, kaempferol, and in comparison to respective aglycons, thus indicating the importance structure–activity relationship. A lack of correlation was found between the IC_50_ of phenolic acids, low-molecular-weight phenolic metabolites, and their E_pa_ values. In contrast, weak positive correlations were found between the IC_50_ of aglicons, 3-*O*-glucosides, 7-*O*-glucosides, and their E_pa_ values provided by the DPV (r = 0.61, r = 0.66 and r = 0.88, respectively). The flavonoids and their glucosides are the most promising ACE inhibitors.

## Figures and Tables

**Table 1:** The ACE inhibitory activity and anodic peak potential of selected phenolic acids (both benzoic and cinnamic acid derivatives) *.

Phenolic Acid	IC_50_ Value (µM)	Equation of the Linear Regression	$\mathrm{Anodic}\;\mathrm{Peak}\;\mathrm{Potential}\;E_{pa\;}$(mV)
*Benzoic acid derivatives*			
Gentisic acid	834.49 ± 9.26 ^g^	y = 0.038x + 18.373	622 ± 8 ^c^
Syringic acid	2756.62 ± 36.34 ^d^	y = 0.007x + 30.428	731 ± 9 ^a^
Vanillic acid	4108.26 ± 48.28 ^c^	y = 0.010x + 7.68549	748 ± 10 ^a^
Protocatechuic acid	6234.53 ± 42.02 ^b^	y = 0.007x + 6.358	417 ± 12 ^e^
*Cinnamic acid derivatives*			
Chlorogenic acid	134.2 ± 2.96 ^j^	y = 0.098x + 36.822	443 ± 9 ^d^
p-Coumaric acid	314.89 ± 9.80 ^i^	y = 0.022x + 43.041	672 ± 10 ^b^
Sinapic acid	493.11 ± 6.14 ^h^	y = 0.038x + 31.262	618 ± 7 ^c^
Ferulic acid	979.58 ± 13.55 ^f^	y = 0.019x + 31.290	611 ± 6 ^c^
Caffeic acid	9105.1 ± 58.2 ^a^	y = 0.004x + 15.401	397 ± 8 ^e^

Data are expressed as means ± standard deviation, *n* = 6. Means in a column labelled by the different letters are significantly different (*p* < 0.05) based on the one-way analysis of variance (ANOVA). * The IC_50_ of the control compounds, such as reduced glutathione (GSH) and captopril, was 41.68 ± 3.45 and 0.00586 ± 0.00004 µM.

**Table 2:** The ACE inhibitory activity and anodic peak potential of selected flavonoids and their glucosides *.

Compound	IC_50_ Value (µM)	Equation of the Linear Regression	$\mathrm{Anodic}\;\mathrm{Peak}\;\mathrm{Potential}\;E_{pa\;}$(mV)
Quercetin	120.54 ± 1.56 ^m^	y = 0.322x + 11.355	330 ± 4 ^g^
Rutin (quercetin-*O*-3-rutinoside)	59.29 ± 0.48 ^t^	y = 0.383x + 27.285	389 ± 4 ^g^
Hyperoside (quercetin -3-*O*-D-galactoside	157.24 ± 1.45 ^n^	y = 0.185x + 20.990	307 ± 3 ^h^
Luteolin	77.10 ± 2.55 ^s^	y = 0.242x + 31.364	407 ± 5 ^f^
Luteolin-4′-*O*-glucoside	88.93 ± 1.68 ^r^	y = 0.236x + 29.039	828 ± 5 ^b^
Luteolin-7-*O*-glucoside	61.50 ± 1.62 ^t^	y = 0.207x + 37.282	445 ± 3 ^e^
Apigenin	336.81 ± 3.72 ^d^	y = 0.080x + 22.954	810 ± 6 ^c^
Apigenin-7-*O*-glucoside	218.44 ± 2.53 ^g^	y = 0.080x + 22.954	841 ± 6 ^ab^
Kaempferol	131.95 ± 2.47 ^k^	y = 0.121x + 34.008	394 ± 4 ^g^
Kaempferol-3-*O*-glucoside	110.27 ± 3.56 ^o^	y = 0.113x + 37.540	359 ± 3 ^fg^
Kaempferol-7-*O*-glucoside	97.70 ± 2.08 ^p^	y = 0.122x + 38.101	419 ± 6 ^f^
Naringenin	390.75 ± 3.96 ^a^	y = 0.079x + 19.287	819 ± 7 ^bc^
Naringenin-7-*O*-glucoside	357.17 ± 1.99 ^c^	y = 0.109x + 11.033	830 ± 8 ^ab^
Cyanidin	176.22 ± 2.38 ^j^	y = 0.142x + 25.065	295 ± 3 ^h^
Cyanidin-3-*O*-glucoside	185.38 ± 2.58 ^i^	y = 0.137x + 24.696	373 ± 4 ^g^
Delphinidin	247.72 ± 1.85 ^f^	y = 0.135x + 16.657	259 ± 3 ^i^
Delphinidin-3-*O*-glucoside	208.78 ± 1.33 ^h^	y = 0.139x + 21.001	453 ± 4 ^e^
Pelargonidin	292.68 ± 3.40 ^e^	y = 0.123x + 13.883	228 ± 5 ^j^
Pelargonidin-3-*O*-glucoside	376.88 ± 2.61 ^b^	y = 0.116x + 6.433	473 ± 5 ^d^

Data are expressed as means ± standard deviation, *n* = 6. Means in a column labelled by the different letters are significantly different (*p* < 0.05) based on the one-way analysis of variance (ANOVA). * The IC_50_ of the control compounds, such as reduced glutathione (GSH) and captopril, was 41.68 ± 3.45 and 0.00586 ± 0.00004 µM.

**Table 3:** The ACE inhibitory activity and anodic peak potential of low-molecular-weight phenolic acid metabolites *.

Phenolic Acid	IC_50_ Value (µM)	Equation of the Linear Regression	$\mathrm{Anodic}\;\mathrm{Peak}\;\mathrm{Potential}\;E_{pa}$(mV)
3-hydroxyphenylacetic acid (3-HPAA)	678.86 ± 7.70 ^f^	y = 0.052x + 14.971	883 ± 11 ^b^
4-hydroxyphenylacetic acid (4-HPAA)	1390.67 ± 10.18 ^b^	y = 0.027x + 12.730	815 ± 12 ^c^
3,4-dihydroxyphenylacetic acid (3,4-DHPAA)	672.82 ± 4.93 ^f^	y = 0.048x + 18.041	432 ± 6 ^g^
4-hydroxybenzoic acid (4-HBA)	788.32 ± 11.08 ^d^	y = 0.039x + 18.546	961 ± 11 ^a^
3-(3,4-dihydroxyphenyl)propionic acid (3,4-DHPPA)	612.04 ± 6.51 ^g^	y = 0.054x +16.705	218 ± 3 ^h^
3-(2-hydroxyphenyl)propionic acid(2-HPPA)	932.92 ± 6.42 ^c^	y = 0.044x + 8.952	781 ± 9 ^d^
3-(4-hydroxyphenyl)propionic acid (4-HPPA)	490.65 ± 4.45 ^h^	y = 0.053x + 24.143	664 ± 8 ^e^
3-(2,4-dihydroxyphenyl)propionic acid(3-2,4-DHPPA)	744.66 ± 8.33 ^e^	y = 0.048x + 14.033	659 ± 8 ^e^
3-(4-hydroxyphenyl)propionic methyl ester (MET)	726.02 ± 5.71 ^e^	y = 0.045x + 17.692	574 ± 5 ^f^
2-(3-methoxy-4-hydroxyphenyl)acetic acid (homovanillic acid) (HVA)	1460.54 ± 10.80 ^a^	y = 0.019x + 10.253	643 ± 5 ^e^

Data are expressed as means ± standard deviation, *n* = 6. Means in a column labelled by the different letters are significantly different (*p* < 0.05) based on the one-way analysis of variance (ANOVA). * The IC_50_ of the control compounds, such as reduced glutathione (GSH) and captopril, was 41.68 ± 3.45 and 0.00586 ± 0.00004 µM.

## Data Availability

The original contributions presented in this study are included in the article/Supplementary Materials. Further inquiries can be directed to the corresponding author.

## Supplementary Materials

The following supporting information can be downloaded at: https://www.mdpi.com/article/10.3390/metabo15070443/s1, Figure S1: The IC_50_ of ACE inhibitory activity of selected phenolic acids, flavonoids, their *O*-glucosides, and low-molecular-weight phenolic metabolites.
