# Supplementary figures and images for: Angiotensin-Converting Enzyme Inhibitory Activity of Selected Phenolic Acids, Flavonoids, Their O-Glucosides, and Low-Molecular-Weight Phenolic Metabolites in Relation to Their Oxidation Potentials
Source: Metabolites. 2025 Jul 1;15(7):443. doi: 10.3390/metabo15070443 (PMC12298876; doi:10.3390/metabo15070443)

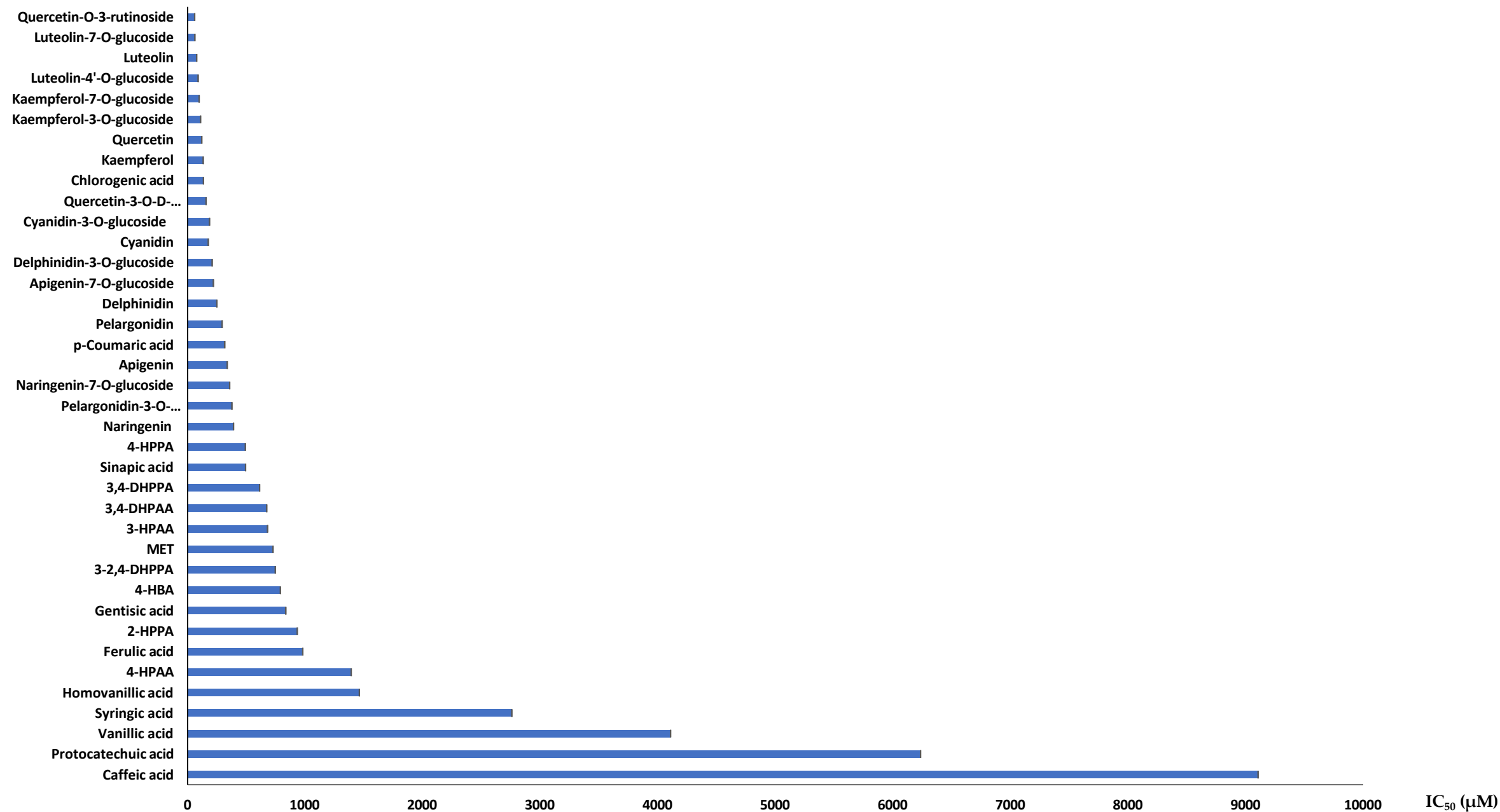

Supplement: Supplementary file 1 [file metabolites-15-00443-s001.zip › metabolites-3673727-supplementary.pdf]
